# Correction: Why are listeners sometimes (but not always) egocentric? Making inferences about using others’ perspective in referential communication

**DOI:** 10.1371/journal.pone.0259617

**Published:** 2021-11-01

**Authors:** J. Jessica Wang, Natalia Ciranova, Bethany Woods, Ian A. Apperly

The following information is missing from the Data Availability statement: Trial level data and the R code used in the analyses, and response time analyses from the current study, can be accessed on the Open Science Framework database (https://osf.io/4ax27).
