# Supplementary material for: Why are listeners sometimes (but not always) egocentric? Making inferences about using others’ perspective in referential communication
Source: PLoS One. 2020 Oct 26;15(10):e0240521. doi: 10.1371/journal.pone.0240521 (PMC7588066; doi:10.1371/journal.pone.0240521)
Supplement: S1 File — (DOCX) [file pone.0240521.s001.docx]

**Supplementary material: RT analysis**

**Descriptive statistics**

| **E1** |  | with-example | | | | without-example | | | |
| --- | --- | --- | --- | --- | --- | --- | --- | --- | --- |
|  |  | experimental | | | | | | | |
|  | **RT** | **2971** | | | | **2943** | | | |
|  | (SD) | 191 | | | | 253 | | | |
|  |  | control | | | | | | | |
|  | **RT** | **2888** | | | | **2941** | | | |
|  | (SD) | 166 | | | | 165 | | | |
| **E2** |  | self-explicit | | | | self-not-explicit | | | |
|  |  | other-explicit | | other-not-explicit | | other-explicit | | other-not-explicit | |
|  |  | experimental | | | | | | | |
|  | **RT** | **3157** | **3050** | | **3128** | | **3103** | |  |
|  | (SD) | 207 | 298 | | 282 | | 322 | |  |
|  |  | control | | | | | | | |
|  | **RT** | **3090** | | **3120** | | **3094** | | **3028** | |
|  | (SD) | 185 | | 225 | | 248 | | 240 | |

**Experiment 1**

Linear mixed effects models were fitted to response times data using the lmer() function from the lme4 package in R [29].

The maximal model for response times included intercepts from both random effects and slopes for all within-unit factors (i.e., condition | participant, magnitude of common ground | participant, condition*magnitude of common ground | participant, condition | grid image, task instruction | grid image, condition*task instruction | grid image). The fitted model for response times did not contain the task instruction | grid image and condition*task instruction | grid image slopes.

An effect of condition was found in response times (control < experimental), with an interaction between condition and magnitude of common ground. Two sets of linear mixed effects models revealed no significant difference among the magnitude conditions within the control condition (*p* = .656) and experimental condition (*p* = .156). Post-hoc t-tests showed no significant difference between the experimental and control condition in the 3 condition (*p* = .491), a marginally significant difference in the 5 condition (*p* = .013). Significance level was set to 0.125 after Bonferroni corrections. The response times on the experimental trials were faster than those on the control trials in the 7 condition (*p* = .002) and 9 condition (*p* = .003).

|  | β | | SE | | χ2 | | df | | p | | |
| --- | --- | --- | --- | --- | --- | --- | --- | --- | --- | --- | --- |
| **Response time** | | | | | | | | | | |  |
| condition | 69.84 | 19.34 | | 9.85 | | 1 | | **0.002** | |  |  |
| instruction | -40.76 | 42.51 | | 0.91 | | 1 | | 0.340 | |  |  |
| mag | 32.18 | 70.07 | | 0.21 | | 1 | | 0.647 | |  |  |
| instruction*condition | 23.98 | 34.87 | | 0.47 | | 1 | | 0.494 | |  |  |
| condition*mag | 123.09 | 48.74 | | 5.73 | | 1 | | **0.017** | |  |  |
| instruction*mag | -31.30 | 45.48 | | 0.47 | | 1 | | 0.495 | |  |  |
| condition*instruction*mag | -80.85 | 87.86 | | 0.84 | | 1 | | 0.360 | |  |  |

**Experiment 2**

The fitted response times model contained intercepts for all random effects, and slopes for condition | participant, condition | grid image, condition*self-perspective-inhibition | grid image, condition*other-perspective-use | grid image, condition | reward, other-perspective-use | reward, self-perspective-inhibition | experimenter, and self-perspective-inhibition*other-perspective-use | experimenter.

An effect of condition was found in response times (control < experimental).

|  | β | SE | χ2 | df | p |
| --- | --- | --- | --- | --- | --- |
| **Response time** | | | | | |
| self | -46.41 | 62.75 | 0.54 | 1 | 0.463 |
| other | -33.12 | 44.55 | 0.55 | 1 | 0.458 |
| condition | 46.43 | 19.95 | 4.04 | 1 | **0.044** |
| self*other | -25.10 | 102.15 | 0.06 | 1 | 0.812 |
| self*condition | 35.50 | 31.62 | 1.24 | 1 | 0.266 |
| other*condition | -31.23 | 30.31 | 1.04 | 1 | 0.307 |
| self*other*condition | 104.74 | 56.75 | 3.38 | 1 | 0.066 |

**Replication**

Main effect of condition on response time was found in both experiments (control < experimental).
