# Supplementary material for: Why are listeners sometimes (but not always) egocentric? Making inferences about using others’ perspective in referential communication
Source: PLoS One. 2020 Oct 26;15(10):e0240521. doi: 10.1371/journal.pone.0240521 (PMC7588066; doi:10.1371/journal.pone.0240521)
Supplement: S1 Appendix — (DOCX) [file pone.0240521.s002.docx]

**Appendix A**: complete list of critical instructions

| Nudge the large balloon one slot down |
| --- |
| Nudge the large present one slot up |
| Nudge the short plant one slot up |
| Nudge the narrow bottle one slot up |
| Nudge the narrow cup one slot up |
| Nudge the small ball one slot up |
| Nudge the short jar one slot down |
| Nudge the tall lamp one slot down |
| Nudge the tall stool one slot up |
| Nudge the thick cushion one slot down |
| Nudge the thick folder one slot down |
| Nudge the thin book one slot up |
| Nudge the thin towel one slot up |
| Nudge the wide car one slot down |
| Nudge the small tin one slot down |
| Nudge the wide vase one slot down |

The critical sentences only involved up/down directions to avoid err due to confusion over left/right. Overall there were an equal number of instructions involving each of the 4 possible directional words.
