# Supplementary material for: Why are listeners sometimes (but not always) egocentric? Making inferences about using others’ perspective in referential communication
Source: PLoS One. 2020 Oct 26;15(10):e0240521. doi: 10.1371/journal.pone.0240521 (PMC7588066; doi:10.1371/journal.pone.0240521)
Supplement: Attachment — Submitted filename: Response to reviewers.docx [file pone.0240521.s003.docx]

Dear Dr Duran, Dr Yoon, and Dr Galati,

Thank you very much for the constructive and detailed comments. We have made revisions and/or comments under each point that you have made. We feel that the manuscript has improved as a result, and we hope that you would agree.

Sincerely

Jessica Wang, Natalia Ciranova, Bethany Woods, & Ian Apperly

PONE-D-20-01714

Why are listeners sometimes (but not always) egocentric? Making inferences about using others’ perspective in referential communication

Dear Dr Wang,

Thank you for submitting your manuscript to PLOS ONE. After careful consideration, we feel that it has merit but does not fully meet PLOS ONE’s publication criteria as it currently stands. Therefore, we invite you to submit a revised version of the manuscript that addresses the points raised during the review process.

==============================

I carefully read the manuscript myself and asked two reviewers, both experts in perspective-taking and referential communication to evaluate the quality of the manuscript. The reviewers provided what I consider to be excellent and fair critiques, as well as several suggestions for improvement. Based on the reviewers’ comments, their ratings, and my own evaluation of the manuscript, I am pleased to offer you an opportunity to revise and resubmit an updated manuscript for potential publication in PLOS One. Note that this invitation to revise does not constitute a guarantee of eventual acceptance. If you choose to revise the manuscript, I will likely send it to the same reviewers to reevaluate. I also ask that you please provide point-by-point responses to each comment, either describing changes made in response to the comment or explaining why no change was made.

For the content of the reviews, I will not repeat all the various issues as they speak for themselves, but I will focus on a few issues that were particularly salient.

Both reviewers question the theoretical claims that are being made. Reviewer 1 would like to know more about the motivation and background that drove this work and Reviewer 2 wonders what is exactly meant by claims concerning the heart of the motivation, this idea of “full chain of inferences.” In my own reading, I too wanted a clearer accounting of what you exactly meant by the inferential steps that are being examined and a more explicit foreshadowing of how these steps are being manipulated within your paradigm/instructions. It seems that what you mean by “full inferential” (or “full chain of inferences”) is this idea of “other-explicit” and “self-explicit,” that is, a speaker has a unique perspective that needs to be taken into account and the self has a unique perspective that might been to be overridden. In the Introduction, the only inferential step that is emphasized is the “other-explicit.” It is only when the reader gets to the Methods that what is meant by full inferential steps (at least I think) is made clear - and really, it was only when reading through Table 1 and 2 captions that the ideas began to fall into place.

* RESPONSE: We have edited the introduction (p5-6) to set up the inferential steps required early on. We have also edited p8-9 to ensure both inferential steps are spelt out. We have responded to R1 and R2 under their specific comments about the theoretical motivation and the full chain of inferences.

Reviewer 2 also wonders whether their understanding of the results of Experiment 2 - as being a sequential step of inference - is correct, and if so, greater discussion of what this means in terms of cognitive processing is needed.  I share with Reviewer 2 a concern about whether my understanding of the claims is correct and what this means for interpretation. For example, there is a claim repeated in the Discussion that having the full chain of inferences required to use the speaker’s perspective was sufficient to prompt successful ToM-use. But based on the results from Exp 2, I do not quite see how this can be true. Do not the results show that having just an other-explicit component produced a similar pattern of results as having both other-explicit and self-explicit components in the instructions? If so, then having examples with a full chain of inferences is not required (as stated on line 447 and in the Conclusion section). It is useful but not absolutely necessary.

* RESPONSE: We do not think that *only* other-perspective-use is needed, instead, it is the inference associated with using other’s perspective that is commonly overlooked by participants. Therefore we see an improvement when they were prompted with an example of such inference. We have clarified the wording of the ‘chain of inference’ with a direct response to R2.

I should note that when reading Reviewer 2’s point above, it also made me think of how this all relates to the theoretical framing used in the Introduction, namely the section on p. 7 lines 130-145 on Apperly’s three constituent stages. I would like to see how this earlier theoretical framing helps informs the current results. In the Introduction it is stated that previous studies observed egocentric errors at the “use” stage of Apperly’s model, but it seems that something else is being argued here for the current paper. The issue instead is about “calculating” not just others’ mental states (other-explicit) and explicitly recognizing one’s own perspective as being a potential distractor (self-explicit). But I am unclear on these finer theoretical points and how it relates to a full chain of inference.

* RESPONSE: We think that the inferential steps we highlighted in the current study are associated with the ‘use’ stage of the Apperly model, rather than the calculation stage, as the sticking point is not at the point of working out the director’s perspective content, but rather how her perspective needs to be integrated/used in reference resolution. We have now spelt this out more explicitly in the introduction (p8) and discussion (p26).

There were also potential study design concerns that raised red flags for interpretation. For example, Reviewer 1 notes that in the error rate is very high in the instruction condition without examples. Reviewer 1 asks whether the error rate is based on participants' final selection. This question dovetails with Reviewer 2’s astute observation that it is unknown how correct object selection (and what constituted an error) was operationalized. Another red flag brought up by Reviewer 1 is why both object size and color were varied within targets and distractors. I agree that this makes the correct (ToM) selection harder to process and could be contributing to the high error rate. And lastly, Reviewer 2 asks for greater clarification on whether the control and experimental versions were in the same block as this definitely could confound results. Where limitations exist, please be sure to make corrections and/or address them thoroughly in the Discussion.

* RESPONSE: We have responded to each reviewers’ specific concerns under their comments.

Reviewer 2 also makes the excellent point about this issue of the sensitivity of your measures to fully capture the cognitive dynamics that seem to be of interest in this paper. That is, you are interested in how response behavior changes over time. I strongly encourage you to consider adding trial ID as a fixed effect as I agree it would give you much better insight (perhaps error rates drop more dramatically over time in the full inferential sequence instructions than in the partial inferential sequences). But whether you add or not is not a condition for acceptance, but it would be good to have some speculation on this matter in the Discussion.

* RESPONSE: We have attempted the exploratory analysis that includes trial ID as a fixed effect. For E1, a non-conclusive trialID*task instruction interaction was observed (p = .013, BF_01_=1.51, which is in the 0.33-3 range of ‘do not favour either H0 or H1’). For E2, it was not possible to converge the model until we were left with subject as the only random effect. We felt that it was important to ensure that the basic random effects were included, rather than pursue an exploratory analysis at the expense of the basic effects. Although the non-conclusive outcome of the exploratory analysis gives us limited scope for further speculation, we agree that it is worth highlighting the approach to think about the dynamic timecourse of interactions, and we have pointed to the relevant papers on p27.

In conclusion, although there are several major issues that need to be addressed, I hope they can be resolved because the topic is very interesting and I think this work can make an important contribution to the field.

Again, please address every concern raised by the reviewers, as well as my specific comments that follow below. I look forward to seeing your revised version. Thank you. -Nick Duran

MY OWN ADDITIONAL COMMENTS:

Plos One puts great value on data availability and transparency in the code for statistical analysis. Perhaps I missed it, but is this information made available to the reader who might be interested in replication or even checking the statistical models for accuracy? Such data could be made available on an OSF repository ([osf.io](https://eur02.safelinks.protection.outlook.com/?url=http%3A%2F%2Fosf.io%2F&data=02%7C01%7Cj.wang40%40lancaster.ac.uk%7C7f22e18be22f4cce80b908d7c42dbf50%7C9c9bcd11977a4e9ca9a0bc734090164a%7C1%7C1%7C637193573088273617&sdata=i9xv%2F46kZSe3Z0OE%2BhSjAVfngK%2FdcTOcdvhRs%2BvoCQ4%3D&reserved=0)).

* RESPONSE: Our trial level data and R code are available on OSF (URL included in the manuscript)

**Exp 1.**

For clarification, it would be good to integrate into the main prose the exact nature of the manipulations mentioned in the Table 1 and 2 captions.

* RESPONSE: We think the editor was referring to Table 1 and Figure 1 (as opposed to Table 2). We have integrated the text previously only seen in the captions to the main prose (p10).

I would be interested in seeing the specifications within G*Power for determining sample size for Exp 1 and Exp 2. Given statistical models that involve between-subjects factor and interaction effects, both studies seem underpowered. However, I am more than willing to concede that I am making incorrect assumptions about how you determined sample size.

* RESPONSE: We thank the editor for the prompt to re-examine our power calculations. In the process of doing so, we realised that we have set the number of measurements incorrectly (too high for both experiments). We are grateful to have had to chance to make this correction at this point of the process (on p9 and p20). For transparency, we are attaching a file to explain how we derived at the original required sample sizes (power set at 0.95) and how we think our studies are still sufficiently powered after correcting for the number of measurements, and setting power to 0.8 as a commonly accepted value for power. Please see document “power calculations”

What was the original rationale for the control condition in this study? It was eventually dropped from analysis because participants made so few errors, but curious why errors were even expected to begin with?

* RESPONSE: We have clarified the rationale for including a control condition on p10.

p. 12, line 235: Please provide further explanation for why a large number of trials with correct responses were lost to high rates of error. What is the nature of this error?

* RESPONSE: We were referring to the fact that RT data was based on trials associated with correct responses. We have now clarified this point on p15.

p. 13, line 245: what exactly is the contrast coding scheme used?

* RESPONSE: We employed deviation coding, where each level is compared to a grand mean. We have now specified this on p16.

p. 14, line252: what statistical test did you use to do this comparison?

* RESPONSE: We employed the anova function, which is suitable for comparing variance for one or more fitted model objects. Clarification is added to p16.

In Exp 1 Discussion, I could not follow the argument being made about social functioning profiles (around line 287). Is this to make an argument that mere instructions are sufficient for bringing greater awareness/relevance to a director’s perspective? Please clarify in the prose.

* RESPONSE: We have clarified this on p18.

**Exp 2.**

For setting the effect size, you state that you expect a smaller effect size from Exp 1. Why then choose an effect size of 0.6 for Exp 2 when it was set at 0.2 for Exp 1?

* RESPONSE: We thank the editor for raising this question, as it 0.6 was indeed a typo. The calculation that suggested 92 participants was actually based on an assumed effect size of 0.2. We have now corrected this error along with the number of measurements inputted (as explained to an earlier point) on p20.

What was the rationale for fixing the grid size to 8 in Exp 2 when it was allowed to vary across a range for Exp 1?

* RESPONSE: We have clarified on p10 that the varying magnitude was part of another series of studies. The grid size 8 was set to match the complexity of the array in Apperly et al (2010) which was the first computerised director task, and a widely adopted version of the task. We clarified the latter point on p20.

The alternative hypothesis on page 20, starting line 383, is hard to follow and it took me some time to work out. I think what is being claimed is that an ego-centric bias was still compete with responses even with the clearest of instructions. Is this right? And this appears to be evidenced with the more detailed instruction (other- and self-explicit) where the error rate following first correct response is still high (above 0) and does not vary from that much from the less detailed instructions? It would be useful to know which numbers are being compared from Table 2 to make these claims. Also, the introduction of the alternative hypothesis arrives out of the blue. I would recommend introducing the alternative hypothesis earlier as a target for analysis/interpretation (rather than doing it in the Results section), or treat it as a standalone issue/explanation as you do with the section “Replication of Experiment 1.”

* RESPONSE: We take the editor’s point that the alternative account should have featured earlier in the manuscript. We now explained such an account and the rationale behind the floor-level test when we introduced the DVs (p14-15). The statistical tests are now reported in E1’s results section (p17) and E2’s replication of E1 section (p23 for E2), where we also spelt out the mean error rates.

==============================


Review Comments to the Author

Please use the space provided to explain your answers to the questions above. You may also include additional comments for the author, including concerns about dual publication, research ethics, or publication ethics. (Please upload your review as an attachment if it exceeds 20,000 characters)

**Reviewer #1**: This research examines the role of explicit instructions on listeners’ perspective-taking in the referential communication task. When the director’s scene and the listener’s scene differ, the listener needs to consider the common ground rather than taking their privileged ground. Previous studies have shown that listeners are not always successful to take the director’s perspective to identify a target following the director’s instruction. In two experiments, the authors manipulated the types of instructions and tested listeners' perspective-taking. The results showed that listeners made fewer egocentric errors when they were given explicit instructions with examples (E1). Especially, explicit instructions on using the director’s perspective modulated listeners’ performance, but instructions on inhibiting their privileged perspective did not help (E2). Thus, the findings showed that listeners can take the director’s perspective when they are prompted with appropriate instructions.

I have a few comments and questions below.

First of all, I was not fully convinced with the motivation of this research. It is not quite surprising to see participants’ performance improved with explicit instructions. In introduction, it needs to build theoretical motivation and background that drove this research.

Especially, providing explicit instructions in this research may lack ecological validity. In everyday life, we are not told about perspective differences between partners. We naturally notice it and integrate that information in language processing. I think it is OK to use manipulations that lack ecological validity, but it needs justification. In that sense, it would be helpful to read about theoretical motivations about this research question/manipulations in introduction (and theoretical implications in discussion as well).

* RESPONSE: We’ve spelt out more explicitly our motivation to understand the inferential processes embedded in the Apperly (2010) mindreading model, and to account for the high rates of egocentric errors observed in the literature. We have spelt this out further in the introduction (p8-9) and in the discussion (p26). We have also added a comment about using the current findings to understand real life variability in mindreading (p27).

Secondly, in this sort of experimental paradigm in which perspective-taking is tested, researchers have manipulated only the “size” of critical items, the color remained the same (Ryskin et al., 2015). However, in this research, for example in Fig 1, the colors of the three presents are different and I think this is problematic. People use color adjectives all the time, and especially in Fig 1, speakers would not describe the target item using a size adjective. They would be highly likely to describe it using a color adjective (e.g., the purple present). If so, when participants heard the size adjective, it is just difficult for them to process. Why would you use a difficult size adjective when you can simply use a color adjective? I was wondering if all items were like in Fig 1 (critical items with different colors)? Or do critical items in Fig 1 and Table 1 happen to have different colors? It needs to be clarified.
* RESPONSE: We appreciate the point that colour adjectives are used frequently. However, we do not think that participants incurred observable difficulty in processing size adjectives while colours varied. This is evident in the ceiling-level performance in the control condition, where the director delivered the same verbal description as she did in the experimental condition, therefore requiring participants to consider the size (or height, width, thickness) contrast when other aspects of the objects varied.

Thirdly, the results are quite straightforward, but I was surprised by a high rate of errors that participants made especially in the condition without examples (E1 and the comparable condition in E2). Was it based on their final selection? Then, this means that more than half of the time, participants made egocentric errors in E1 (without-example). I understand that listeners consider egocentric knowledge in the middle of processing but it’s surprising that they made a final selection more than half of the time based on their egocentric knowledge. It would be great to clarify the dependent measure and I also wonder if there was a procedure to confirm that participants understood the experimental setting (e.g., what the green background indicated…).

* RESPONSE: the responses were taken at first selection. We made this explicit to participants, and asked them to carefully consider their choices before making a mouse click. We have now clarified this point on p13. From our experience with the paradigm, participants very rarely have trouble understanding the premise that the director cannot see objects placed in slots with green backgrounds. We do take care to show participants the director’s view of the grid to minimise the possibility for misinterpretation.

Note, the high egocentric error rate is very similar to that reported by Apperly et al. (2010) using a similar paradigm and instructions. While the error rate is high, it is far above the level expected (100% errors) if all participants had misunderstood or failed to follow the instructions. Moreover, as can be seen in the pirate plots, the average comprised a broad distribution of errors across participants. In contrast had participants simply varied in their comprehension we would have expected a bimodal mixture of participants who performed either close to floor or close to ceiling.

Regarding the dependent measure, in the results, the authors analyzed the response time (pg. 14) as well as other measures (egocentric error, # of trials to first correct response, and error rate following first correct response), but descriptive stat of response time was not reported.

* RESPONSE: We thank the reviewer for pointing this out – we had omitted the response time data by mistake. It has now been added to the supplementary materials on OSF, as originally intended.

Minor points:
In E2, the authors included grid image, reward, and experimenter in the model. To make this consistent across experiments, it needs to be done in E1 as well. (Or if it is already done, it needs to be reported.)
* RESPONSE: This has now been clarified on page 16.

In Table 1, the condition labels were not clear. I had to read back and forth to understand each condition. If it can be clarified, it would be helpful.
* RESPONSE: We have added further clarifications in the caption, and added labels to the table and colour coded the experiments.

**Reviewer #2**: Summary: Across two experiments, the authors examine the effect of instructions on the distribution of egocentric responses in the "director task". The findings suggest that instructions about the inferential steps of assessing the director's perspective reduce the egocentric errors. Experiment 2 clarifies that it is the instructions' emphasis on the use of the task partner's perspective that drives this effect.

I have reviewed this article before at another journal. In this review, I focus my assessment on the extent to which the authors addressed my previous comments adequately in this submission. As I noted in my previous review, overall, I appreciate the novelty of the study in disentangling the effect of instructions to inhibit one's perspective vs. to use the task partner's perspective.

The authors effectively addressed one of my earlier comments about the theoretical framing (namely, about instructions previously being framed as motivating listeners to make inferences about the speaker’s perspective). Still, a number of my previous comments persist, including methodological and analytical points that could have been easily clarified.

I present these pending methodological issues next:

1. Please be explicit about whether participants saw both the control and experimental version of an otherwise identical display (i.e., whether they saw both displays shown in Figure 1). If so, I assume that critical and experimental versions were not in the same block, since this contrast could highlight the disparity between self and other perspectives and could confound the results. In either case, please specify how items were distributed across trials and blocks.
* RESPONSE: Participants did see both control and experimental variations of the same base grid, but each counterpart of an image was placed at least a full block away. We have clarified this point on page 13.

2. The distinction between conditions (control, experimental) is opaque upon first mention (p. 9), and is not fully described until p. 11. Please establish early on that these terms have to do with the absence vs. presence of a distractor in the trial.
* RESPONSE: We have now clarified this point on page 10.

3. It’s not clear initially, on p. 9., what the "magnitude of common ground" means (“3, 5, 7, 9”). On p. 13 the authors suggest that these values refer to "slots", but whether they are slots in common ground or NOT in common ground remains unclear. (Figure 1 suggests that it's the latter, seeing that there are 5 occluded and 11 open slots.) Please clarify.
* RESPONSE: We have now clarified this point on page 10, where the magnitude of common ground was first mentioned.

4. It's not clear what would constitute egocentric errors in the control condition (p. 12, line 222). If the correct selection is the same object from both the participant's and the director's perspective, I don't follow how there could be an egocentric error to even yield the "2 egocentric errors” observed in the control condition across the two studies. Did the authors count the selection of the irrelevant object as an egocentric error (under the rationale that it is at the same location as the distractor/egocentric item of a corresponding display)? That doesn’t seem to be a felicitous interpretation of “egocentric”.
* RESPONSE: We agree with the reviewer that in the control condition there is nothing to critically assess egocentrism. The ‘egocentric error’ was indeed calculated as selection of the irrelevant object, which was designed to provide a baseline of erroneous selection compared to egocentric errors. We have edited the wording on page 14 to clarify this point.

5. Please specify how correct object selection and response times were operationalized. Was response time based on the first click on the computer screen? on the release of the drag-and-drop gesture? on the final object selection or drag-and-drop release? All of these definitions are plausible. What counted as correct object selection: clicking anywhere in the cell of that object or at a more circumscribed area? Please clarify how data was collected from participants' interaction with the displays.
* RESPONSE: We have clarified these points of ambiguity on page 13.

Some persisting theoretical points:

1. The authors refer to the instructions as describing the "full chain of inferences". What is that chain of inferences exactly? Is the chain of inferences: “Can she see my largest present? If yes, select. If not, which is the next largest present?” Or something else? In the Conclusion section, the authors present two inferences: “1. [Listeners] should not use their own perspective to resolve reference, 2. the specific ways in which a speaker’s perspective constrains reference.” Is this the chain of inference? If so, framing this as a chain suggests a sequential order. And if that’s the claim, the authors need to be explicit about how their results from Experiment 2 support it.
* RESPONSE: The two inferential steps we have discussed in the Conclusion section are indeed the ‘chain’ we refer to. We do not think that the two steps of inferences need to occur sequentially. As we discussed in Experiment 2, merely taking the self-inhibition step could have led to successful reference resolution. Therefore the inferential process needs not to be sequential. However, the manipulation we employed in Experiment 1 did follow a consistent order: first we told participants not to use their own perspective, and then we told them how to use the director’s perspective. Therefore in this case a chain does exist and implies the sequential order. We have clarified this point in the introduction (p8-9), the discussion section of E1 (page 19) and the general discussion section (page 24).

2. In this submission it’s not as clear what the rationale is of choosing the 3 DVs (overall rate of egocentric errors, number of trials to first correct response, and error rate following first correct response). The inclusion of the two latter metrics suggests that the first correct response reveals something important about the listener’s cognitive dynamics throughout the experiment. But the authors don’t explain how and why. Much later in the paper (p. 20, line 383-386) the authors suggest that they use these measures to test the alternative hypothesis that the instructions fully clarified what participants should do. The connections between the choice of these measures, the authors’ preferred hypothesis, and this alternative hypothesis need to be clarified.
* RESPONSE: We have responded to a similar concern raised by the editor, and have now clarified this point on p14-15.

As I recommended previously, instead of or in addition to these coarser-grained DVs, the authors could include trial id as a fixed effect in their models. Such an exploratory analysis could offer insights about the stabilization of other-centric responding over time under the different instruction conditions.
* RESPONSE: As indicated to the editor, we have attempted the exploratory analysis that includes trial ID as a fixed effect. For E1, a non-conclusive trialID*task instruction interaction was observed (p = .013, BF_01_=1.51, which is in the 0.33-3 range of ‘do not favour either H0 or H1’). For E2, it was not possible to converge the model until we were left with subject as the only random effect. While we agree that this would have been an informative exploratory analysis, the non-conclusive outcome gave us limited scope to take this further.

A related point: the authors suggest that, because the listeners’ error rates were substantially different from 0% after their first correct response, listeners “did not simply grasp what they were supposed to do” after the instructions. I’m not sure what “grasping what they were supposed to do” means here. But as I pointed out before, perspective-taking is not all-or-none; it need not be invariably at floor or at ceiling.
* RESPONSE: We agree that perspective-taking is not all or nothing, and that the test against a floor level performance was to test an alternative account. We have further clarified and reworded this point on p15.

I leave it up to the Action Editor whether the authors should conduct additional analyses to examine at the distribution of egocentric errors over time in finer grain. But given the authors' theoretical points about sub-stages in perspective-taking and chains of inference in resolving reference, I suggest (as I did before) that they consider related work on how perspective-taking choices evolve and stabilize over time, not just within trials but across trials, over the course of an interaction (e.g., see Duran & Dale, 2014; Duran & Dale, 2011; Dale, Galati, et al., 2019; Galati, Dale, and Duran, 2019).
* RESPONSE: we have made some of these consideration in our discussion on p27.


Small points
• p.4: Thanks for unpacking and future-proofing the Hillary Clinton example. Just a typo: “winner” -> “winning”
• p. 12, line 222: “excluded prior analysis” -> “excluded prior to analysis”
• Please replace R and lmer syntax with words in the text (namely, avoid | and * ).
• Footnote 3: incentivises -> incentives

* RESPONSE: all of the above have all been corrected.
• The two Experiments don't report results consistently. E2 includes Bayes factors, but E1 does not. Please make them consistent.
* RESPONSE: We reported Bayes factors for all null effects of theoretical interest. There was no null effect in E1 to report, hence the absence of Bayes factors. We have corrected all the other points in the manuscript, and we’d like to thank the reviewer for catching these errors.

* ADDITIONAL NOTE: We have replaced the image of the director, as we have lost touch with the actor whose image was used in the experiment. The new images show an actor who have signed consent form for their image to be used in academic publications, as explained on p9.
