# Supplementary material for: Why are listeners sometimes (but not always) egocentric? Making inferences about using others’ perspective in referential communication
Source: PLoS One. 2020 Oct 26;15(10):e0240521. doi: 10.1371/journal.pone.0240521 (PMC7588066; doi:10.1371/journal.pone.0240521)
Supplement: Attachment — Submitted filename: Response to Reviewer.docx [file pone.0240521.s004.docx]

**Response to Reviewers**

Dear Dr Duran, Dr Yoon, and Dr Galati

We’d like to thank you and the reviewers for the additional comments. We have made further revisions to the manuscript, and responded to each point raised by the reviewers. We hope that you would find this satisfactory. Please note that the line and page numbers cited here corresponds to the tracked-change version of the manuscript.

Sincerely

Jessica Wang, Natalia Ciranova, Bethany Woods, & Ian Apperly

Thank you for this resubmission and the obvious efforts in addressing the comments and concerns from the first round of reviews. I believe the manuscript to be much improved because of these efforts, a sentiment shared by the reviewers.

As you can see from the new reviews - which I thank the reviewers for doing with such care - there are still issues to be resolved. Many of these revolve around a theoretical framing that needs to be further clarified and strengthened. There is no requirement here to justify the importance of your theoretical stance, but merely to provide enough detail to remove ambiguity in how your conclusions relate to your data and a larger literature. Your manuscript is strong on all of PLOS ONE’s publishing criteria, except for one: "Conclusions are presented in an appropriate fashion and are supported by the data.” This is not a seriously damning weakness, but a few claims/conclusions need to be tempered given plausible alternative explanations and a referential communication task that has been questioned by others for its ability to genuinely capture perspective-taking behaviors in richer communicative scenarios. The suggestions for how to temper are provided by reviewers. I also urge you to seriously consider other suggestions for how to better draw out the novelty of your findings and their implications, as well as where greater precision in your interpretations could be made. 
 
As before, please address every concern raised by the reviewers by directly communicating what change was made to your manuscript because of the comment, or justification for why you did not choose to make a change.  I look forward to seeing your revised version. This paper should make a strong contribution once all points are addressed. Also know that I am committed to expediting this manuscript to the next stage assuming the necessary changes have been made.

Sincerely, Nick Duran

Reviewer #1: This is a resubmission of work investigating the role of explicit instructions on ToM (taking the partner’s perspective) in a referential communication task.
My main concern in my previous review was related to the theoretical motivation/implication. The authors have edited the manuscript, and Experiments are now more clearly motivated. The manuscript is improved by the more detailed descriptions of the experimental manipulations and analyses in the paper.

I believe the paper is much clearer now although some issues could still be made and I have a few remarks on the revised manuscript.

- Now I understand the theoretical motivation better, but the paper downplays the literature that has shown that listeners readily take the partner’s perspective in language comprehension (Hanna et al., 2003; Brown-Schmidt, 2009, etc.). There have been theoretical debates between the two groups on listeners’ ability to use common ground (Barr, 2008; Keysar et al., 1998; 2003 vs. Hanna et al., 2003; Hanna & Brenna, 2007; Brown-Schmidt, 2009); While the authors described only one side of the literature, there is a great amount of work that demonstrated that listeners could quickly identify the target based on the speaker’s perspective (Hanna et al., 2003; Brown-Schmidt, 2009; Ryskin et al., 2015, etc). I think it’s fair to introduce both sides of the literature in the introduction.

While the current work seems to be more consistent with Barr and Keysar’s claim and at odds with the results of the work by the Tanenhaus group, Arperly’s theory and the current results may contribute to reconciling the two seemingly contradictory claims. I wonder if the authors have thought about this point and if so, include it in the discussion. I bet a wider range of audiences will be interested.

**Response**: The contrasting theoretical positions were downplayed because the current study doesn’t directly address the debate, but we do mention both sides of the argument in lines 69-72 on p4. We appreciate that our findings may have implications for the debate, therefore we have now included a wider discussion point on this in lines 637-648, on p29-30.

- One thing I’d like to comment is that in the current study, listeners benefitted from explicit instructions, but without explicit instructions with examples, they fail to take the partner’s perspective 40-60% of the time (in E1 and E2). Even with this failure, it may not mean that listeners are not able to take the partner’s perspective; they just don’t do without specific motivation or conversational goals. In Yoon, Koh, & Brown-Schcmidt, (2012), the rate of perspective-taking varied depending on the conversational goals (instruction vs. request). Like this, participants in the “without-example” condition might be able to do so as in the explicit instruction conditions, but they might not be motivated enough – which is one of the disadvantages of non-interactive referential communication tasks (compared to interactive version). The claim in the manuscript may need to be tempered.

**Response**: We have now added a discussion point about motivation to the general discussion (lines 632-637, on p29).

- pg, 12, among 96 fillers, 24 contained scalar adjectives, and 14 contained non-scalar adjectives. I guess 58 fillers were bare noun phrases (E.g., bowl). This needs to be clarified. Along with this, I have a question regarding the fillers of scalar adjectives. In Figure 1 and Table 1, I do not see any size-contrast filler items. The only size-contrast item is the critical target item (e.g., torch, present). How were scalar adjectives used to refer to filler items? Listeners are sensitive to the use of adjectives; if speakers use an unnecessary adjective, their comprehension processing might be significantly affected (e.g., when there is only one bowl, and the speaker says “move the small bowl…”).

**Response**: We thank the reviewer for querying about this point, as we had initially miscounted the number of adjectival phrases, which has now been corrected. The information requested has now been added (lines 260-265 on p13).

- In Table 2, please include descriptive statistics of the control conditions. I understand the authors only analyze the critical condition in the model, but it will be helpful to understand the entire data.

**Response**: The full RT data are already included in the supplementary data file. Since it was not possible to commit egocentric errors on the control trials, the table would have comprised of 0s throughout.

- in abstract and in the intro (pg. 4), the phrase “healthy adults have little trouble understanding others’ mental states” is confusing; does it mean they do not have trouble understanding that the partner can have “different” mental state or understanding the exact different view of the partner? For example, one can know that the partner can have a different view without knowing what exactly it can be vs. one can know exactly what the partner has in his view. It needs to be clarified.

**Response**: we have now clarified this point in lines 29-30 on p2.

- pg. 9, Experiment 2 further investigated whether both prompts are required, and whether they need to be given sequentially. --> the later one was not tested.

**Response**: Indeed, this was not our primary research question. However, as our results indicate that not both prompts are required, it also implied that the prompts do not need to be given sequentially. The explicit sign-posting to the sequential presentation question was requested by Reviewer 2.

- In Methods, were all participants native English speakers?

**Response**: Unfortunately this information was not systematically recorded at the time. However, our recruitment criteria was “good English comprehension” and that no participants showed difficulty in following the control or filler trials. Therefore we are satisfied that our findings cannot be attributed to poor English comprehension.

- pg. 26, line 564, “very few listeners spontaneously inferred…” This sentence is not consistent with data in E1 and E2. In Figures 2 and 3, many participants successfully take the partner’s perspective even in the “without-example” condition.

**Response**: we have toned down the wording here (now line 623 on p29).

Reviewer #2: I have carefully read this revised manuscript, which I find considerably improved. You have addressed a number of my previous comments effectively, especially in terms of clarifying methodological points (e.g., how you operationalized correct responses and RTs, the rationale of your different DVs, how control and experimental trials were distributed, etc.).

I find that a few theoretical points can still be clarified further.


1. I appreciate your clarifications about the two inferential steps you identify as necessary for completing the director task successfully: (1) participants must ensure they are guided by the director’s perspective rather than their own (i.e., must inhibit their own perspective), 2. participants work out precisely how the director’s perspective constrains reference. However, I find that more work is needed to clarify these inferential processes further and strengthen the theoretical contribution of the paper.

First, I invite you to be more explicit about what step #2 involves in this director task. Specifically, I encourage you to be more specific about what you mean by “working out” how to use the director’s perspective. Is step #2 the recognition that the director cannot see items in a blocked shelf *AND* that, as a result, their intended referent of a scalar expression (e.g. “the short torch”) can be different from the participant’s?

**Response**: We have now clarified this point in lines 164-175 on P8-9.

Second, by extension, I invite you to be more explicit about how this “working out” of using the partner’s perspective can look like in other contexts involving perspective taking. Doing so can help clarify the connections between these findings and the more generalizable account you espouse, namely, Apperly’s model of mindreading (see point #2).

**Response**: We now comment on this in line 177-188 on p9.

Third, in your response letter, you noted that you don’t consider these steps to be sequential, even though they happen to appear in sequence in the instructions. But this isn’t clearly discussed in the paper. In fact, the language used throughout the manuscript (“steps”, “inferential steps”, “chain of inference”) does suggest sequential steps. Please clarify. This is especially important because steps #1 and #2 together can result in a single heuristic for doing the task (e.g., “skip all blocked shelves”).

**Response**: In E1, the two inferential steps were indeed presented in a sequential order, therefore we feel that it is correct to use wordings such as ‘steps’ and ‘chain’ to reflect this manipulation. We feel that we have spelt out this distinction in the rationale for E2 (line 204-206 on p10). The possible heuristics have also been specified (line 418-424 on p20). We feel that further emphasis on the sequential point would detract from the main focus of the study, as we did not specifically set out to test whether the inferences need to be carried out sequentially, rather we tested whether both steps were necessary.

2. It’s not clear how the findings inform Apperly’s model of mindreading. In the Introduction you describe the two steps of the use stage of perspectival information (lines 158-165). In the Discussion you argue that your study identifies these two sub-processes of the use stage of mind reading (line 549). There’s circularity in this framing.

**Response**: we have now clarified that the aim here is to understand sub-stages of the use stage in Apperly’s model (line 184-188 on p9) so that it is consistent with the statement in the discussion (line 608-611 on p28).

The most novel contribution of this work is the finding that highlighting step #1 with instructions (“inhibit your own perspective”) does not support perspective-taking performance, whereas highlighting step #2 with instructions does. This take-home message is lost a bit. This is in part because whenever the results of Experiment 1 are reported there is emphasis on the fact that that instructions with the “full chain of inferences” were required for successful perspective use. I suggest tempering these statements in your General Discussion, seeing that in Experiment 2 you actually find that an example with the full chain of inferences was not in fact required for successful performance.

**Response**: We have tempered the wording in the general discussion to spell this out (line 568-569 on p26-27).

Overall, I recommend highlighting your most notable contributions about the differential benefit of highlighting through instructions these two inferential steps and providing an explanatory account for this finding. That is: why is it that instructions to suppress the egocentric perspective don’t confer a performance benefit? Is it because adopting the egocentric perspective is fairly automatic and cannot be modulated by instructions? Addressing these points can bolster the theoretical contribution of the paper.

**Response**: we have reworked the discussion section in E2 to include such an account (lines 530-544 on p25-26).

3. I don’t get the alternative account that you’re trying to rule out by testing egocentric errors past the first correct response against 0%. You argue that if participants fully understood the instructions through a more elaborated example, then floor-level error rates should be observed. I don’t see how understanding instructions obligates flawless performance. Human behavior is still replete with errors when there is response conflict, even when instructions are understood. What does “understanding instructions” mean? My general stance is that it is perfectly fine to conduct the test you did to establish that the obtained error rates are higher than zero. But I don’t see this alternative account as a plausible one that you need to rule out. If you retain this alternative account, I recommend unpacking why understanding instructions (which is different from applying the instructions) necessitates floor-level error rates.

**Response**: We think that it is important to retain this account, as overly simplistic as it may sound - it is an obvious alternative account to rule out. We have now clarified the wording on ‘understanding instructions’ in lines 320-326 on p16.

Smaller points:

• You have changed your display in Figure 1 but, in the text, you still refer to “presents” rather than “torches” (p. 13).

**Response**: Table 1 indeed presents and refers to ‘torches’, but Figure 1 still presents and refers to ‘presents’.

• Please include in a footnote your explanation about including Bayes factors for null effects of interest.

**Response**: An explanation has now been added to lines 473-480 on p22-23.

• P. 25, bottom: You suggest that instructions “may have provided additional motivation for participants to make the required inference about using the speaker’s perspective…” This might be residual language from an earlier version of your paper. It’s not clear what you mean here about instructions serving as “motivation” or as a ‘sufficient prompt” (a few sentences below, p. 26 top).

**Response**: We have clarified the wordings in lines 595 and 603-605 on p28.

Typos:
• P. 6, line 101: “not participants’ own perspective” --> “not the participants’…”
• P. 6, line 106: “variations of the director task has” --> have
• P. 8, line 166: “how people use mindreading information Apperly’s (2010).” ??
• P. 18, line 369: “who score highly on either autistic or psychotic characteristic”; add “an” or pluralize (“characteristics”)
• P. 20, line 422: “excluded prior analysis” --> “prior to”

**Response**: all corrected, we thank the reviewer for noting these typos.
